# Preparedness to implement national enteral nutritional therapy practice guidelines: An observational study of primary health care institutions in South Africa

**DOI:** 10.4102/phcfm.v14i1.3056

**Published:** 2022-01-31

**Authors:** Nomaxabiso M. Mooi, Busisiwe P. Ncama

**Affiliations:** 1 Department of Nursing, Faculty of Health Sciences, Walter Sisulu University, Mthatha, South Africa; 2 Department of Nursing and Public Health, Faculty of Health Sciences, University of KwaZulu-Natal, Durban, South Africa

**Keywords:** preparedness, enteral nutritional therapy, practice guidelines, implementation, health cre professionals, primary health care, illness

## Abstract

**Background:**

Despite the long-term consequences of malnutrition in hospitalised patients, nutritional practice guidelines for adults, particularly in the recovery phase are rarely implemented in community based primary health care settings.

**Aim:**

This study aimed at assessing the current practice to establish preparedness for effective implementation of the 2016 South African Enteral Nutrition Practice Guidelines for Adults.

**Setting:**

This study was conducted in a district hospital in KwaZulu-Natal, a community health centre, two primary health care (PHC) clinics and one household.

**Methods:**

Non-participant observations were conducted to observe 10 purposefully selected health care professionals involved in nutritional therapy provision to adults, a patient on home enteral nutrition (HEN) and a family caregiver. Content analysis helped identify predominant themes that emerged in the study.

**Results:**

Observation results showed that the national enteral nutritional (EN) therapy practice guidelines were not available in all health care institutions. Health care professionals were not aware of them and the care users confirmed that they attended HEN related follow-up care at institutions that prescribed and inserted their feeding tubes. Two major themes that emerged in this study include positive factors and negative factors that influence implementation of the guideline.

**Conclusion:**

The study identified factors that can have significant influence on the implementation of the national enteral nutritional therapy practice guidelines, a necessary step for changing clinical practice and thus clinical outcomes of patients. The EN/HEN training and the provision of necessary resources are needed to improve the situation. More research on the strategies for the dissemination of guidelines is essential to improve awareness and thus adoption and implementation.

## Introduction

Research has shown that ill patients, especially in the acute phase, suffer extensive catabolism with resultant malnutrition, which continues to be underdiagnosed and managed during the patient’s hospital stay.^1,2,3^ Patients are estimated to be receiving one-third to one-half of the current recommendation for protein delivery, 1.2–2.0 g/kg per day, which increases the risk of malnutrition.^1,2,4^ Regrettably, international studies show an average delivery of 0.6 g/kg per day of protein during the first two weeks following admission. Dhaliwal postulated that these at-risk patients receive only 61.2% energy supply on average, with 74.0% of them not reaching the standard energy requirement.^5^ The increased catabolism, accompanied by iatrogenic underfeeding, and the subsequent malnutrition is closely associated with infections, delayed wound healing, multiple organ failure, prolonged length of stay in hospital, mortality rate, and increase in medical expenses.^3^

Enteral nutritional (EN) therapy has proven effective in providing nutrients for individuals to nutritionally-at-risk ill patients who are unable to meet their nutritional requirements. It is widely used in subacute, rehabilitation, long-term care and home settings.^6^ When implemented appropriately, EN has improved clinical outcomes, reduced infection rates, number of hospital admissions and health care costs.^7,8,9^ Additionally, the development of percutaneous endoscopic gastrostomy (PEG) technique and the shift to primary health care has popularised home enteral nutrition (HEN) globally.^10^ However, wide disparities in the provision of EN across health care levels have been documented, which is a strong motivation for the development of practice guidelines.^11^

Best practice guidelines can ensure that quality care is provided to HEN patients in the community or home setting.^12,13^ Through guidelines, more individuals on enteral feeds are now managed in the community. In the United Kingdom (UK), a 42.78% increase over a 10-year period in patients receiving HEN has been reported, although there is evidence of a yearly increase (20% – 25%) in the number of people on EN. Despite substantial comorbidity of critical illness and malnutrition, nutritional practice guidelines for adults recovering from critical illness are rarely implemented in community based primary health care settings.^14^

South African enteral nutritional therapy practice guidelines (ENPGs) for adults were developed in 2016 in response to calls by international organisations.^15,16,17^ These calls were aimed at supporting countries to act against malnutrition according to their national circumstances and resources, with emphasis on vulnerable groups.^18,19,20^ The guideline developers noted with concern that malnutrition occurs in about 15% – 70% of hospital patients. It was often undiagnosed in about 70% of the patients with 70% – 80% being admitted to hospital already malnourished and discharged without receiving any nutritional therapy.^15,16,21^ The guidelines were designed for nutritional assessment and provision and monitoring of EN delivery in adult patients at public health care facilities. The national guidelines were not confined to hospitalised patients, but from before hospital admission through to discharge. The study, therefore, was to assess the current practice to establish preparedness for the effective implementation of the guidelines in the district hospital (DH) and primary health care (PHC) settings.

## Methods

### Study design

Based on the purpose of the study, non-participant observations were conducted to gain a better understanding of the practice environment and context related to the implementation of the national enteral nutritional practice guidelines for adults. The method made it possible to observe things that routinely escape the awareness of the participant using a different method. Furthermore, the research approach is known to provide a chance to learn things that people may be unwilling to discuss in an interview or state in a questionnaire.^22^ Kawulich^23^ noted that non-participant observation facilitates the maintenance of a sense of objectivity through distance, compared to participant observation.

### Research paradigm

The research is underpinned by deductive interpretivism, in which the researcher aims to understand complex human phenomena and then interpret them.^24^ Combined with content analysis, the approach is suitable for workplace analysis.^25^ The approach blend well with observational research, which is known to enhance contextualisation. This means that whatever the sphere in which the data are being collected, we can only understand events when they are situated in the wider social and historical context,^26^ which is what the current study intended to do.

### Study setting

The study was conducted in a 185-bedded DH in KwaZulu-Natal, a community health centre (CHC), two feeder PHC clinics, and one household. The hospital provides care to trauma and emergency, medical and surgical patients, and has a potential infrastructure for nutrition services that include enteral nutrition to patients in need. The selected district has a total population of 478 537 with high unemployment rate, informal housing, and 93% of the population living below poverty line.^27^ Its geographical layout, the resultant poor roads infrastructure and transportation constraints contribute to the poor access to health facilities. Cross border flow of ill people from Lesotho and Eastern Cape Province further burdens the already strained health services. This demographic profile constitutes the reason for the selection of the district as the study setting. Implementing ENPGs in the selected setting will not only improve the health status of the population but will also improve its socio-economic status.

### Informants

The study targeted all health care professionals involved in the implementation of the ENPGs for adults in the selected setting and adult patients on HEN. The study population comprised 10 health care professionals (HCPs); two doctors, three nurses, three dietitians, two staff from the rehabilitation unit and one patient on HEN and a family caregiver. Purposive sampling was used to select the district, the hospital and health centre. The PHC clinics were recommended by the hospital and the CHC. The household, the patient on HEN and family caregiver were purposefully selected on the recommendation by the referral hospital. Convenience sampling was used to select doctors, professional nurses, dietitians and staff from the rehabilitation unit.

### Inclusion criteria and exclusion criteria

A health care professional had to work in the selected institutions and play a role in the provision of nutritional therapy in adult patients. The basis for the selection of the ill adult patient and family caregiver was on assumed potential to provide rich information regarding the practice owing to the number of years on HEN. The health care professionals dealing with neonatal or paediatric EN or HEN only were excluded.

### Intervention and expected outcomes

This study forms part of a case study aimed at developing a model for the implementation of ENPGs for adults. Examining the practice environment and context were expected to provide the platform for understanding the context in terms of how the guidelines were implemented, to identify any gaps. Therefore, aspects that were observed included guidelines or related documents, implementers, physical environment, as well as interactions and verbal informal conversations that supported the observations. Expected primary outcomes included signs of preparedness to implement guidelines, a conducive environment, culture and supportive context for guideline implementation. Secondary outcomes were clinical outcomes such as nutritional status related hospitalisation, readmissions and mortality.

### Gaining entry and establishing rapport

The researcher brought letters of introduction to ease entry, such as information about the planned length of time in the field and permissions from the provincial department of health, district and the institution. There was no need to meet community leaders, as the institutions were aware of the visit months before the commencement date. Rapport was also enhanced through ‘Hanging out’, which is defined as the process through which the researcher gains trust and establishes rapport with participants. As a result, the researcher was allowed access to sites without formal appointments.^23^ Lincoln^28^ identified that within naturalistic inquiry, the knower and the known are interactive and inseparable. Qualitative research therefore supports the importance of establishing a relationship with participants through building comfort, trust, and ultimately rapport between the participant and the researcher.^29^

### Data collection tool

**FIGURE 1:**
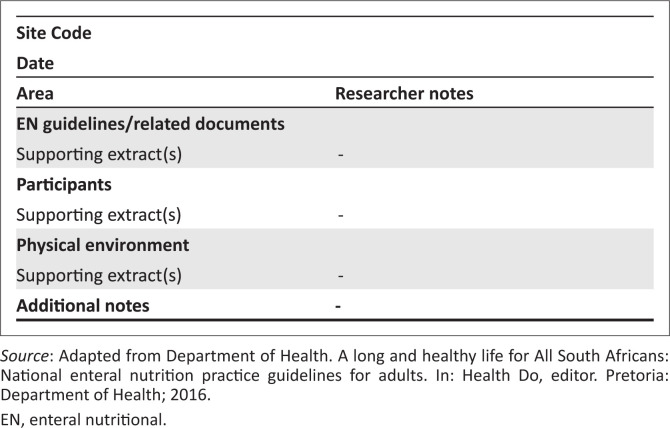
Information observed in relation to the implementation of enteral nutritional therapy guidelines.

As recommended by Merriam and Tisdell,^30^ the researchers adopted an observation checklist from the national EN practice guidelines for adults,^15,31^ (see Figure 1^15^). The checklist was used to record what was observed, which helped to focus on different aspects and types of interactions to help delineate the differences in those activities and institutions.

### Data collection

Following approval from the university ethics committee, the KwaZulu-Natal Department of Health and the respective institutions, data collection was conducted between June and September 2018. The principal researcher accessed an institution a day before the data collection date to get familiar with the surroundings. Arrangements were made through the management of the institution, chief executive officer or operational manger to access the institution and participants. The health care professionals gave consent on the understanding that they would be observed to obtain a better understanding of their nutritional therapy practices. They were not shown the observation checklist used to record for each identified information that is needed, the type of sources used and what was done with the information, in case it biased their normal behaviour. Kawulich^23^ suggested that one should be honest, but not too technical or detailed, in explaining to participants what they are doing.

By using separate checklists for each institution, the participants were observed for two to three visits of 2–4 h each for a period of two weeks, which was considered a sufficient length of time to obtain an accurate assessment of how prepared the institutions were to implement the national guidelines. Apart from the observation checklists, field notes were recorded after each observation session and included observed interactions amongst the professionals and the patients as well as informal conversations with the participants to support the observations, which were also useful to our interpretation of events and contextual information. The field notes and informal conversations were used to more clearly assess the preparedness of the HCPs as compared with what would have been obtained if only structured observational data had been collected. The aforementioned methods used for health facilities also holds for the household. Two days were enough to observe the patient, family caregiver and the surroundings.

### Data analysis

**FIGURE 2:**
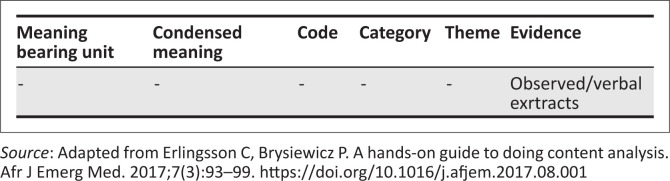
Example of matrix showing how the themes were developed.

Observation notes were transcribed, coded and analysed using deductive content analysis. As stated by Elo and Kyngäs,^32^ deductive content analysis is used when the structure of analysis is operationalised on the basis of previous knowledge; and in this study, analysis was based on what is already known from literature, the knowledge translation theory, and the national EN practice guidelines for adults. Data in the form of text were read thoroughly, coded and then themes were formulated as described by Graneheim et al.^33^ For easy reporting, the themes of the qualitative analysis were arranged under several headings. An example of the analysis matrix showing steps followed in qualitative content analysis as described by Erlingsson and Brysiewicz^34^ are shown in Figure 2.

### Trustworthiness

Contrasted to Guba and Lincoln,^35^ whose goal was trustworthiness, Morse^29^ had suggested that the time has come to return to the terminology of mainstream social science, using rigour rather than trustworthiness. Morse^29^ suggested replacing dependability, credibility, and transferability with the more generally used reliability, validity, and generalisability. In this study, attempts were made through prolonged engagement and persistent observation to achieve internal validity as suggested by Morse.^29^ The other validity procedure that was used was disconfirming the evidence by disclosing categories that were formed a priori in the study, and were then searched in the data for evidence that was consistent with, as McClement et al.^36^ did in their study.

## Ethical considerations

The study was approved by the University of KwaZulu-Natal Human & Social Sciences Research Ethics Committee (HSS/1495/017D) and KZN Department of Health (HRKM413/17), copies of the ethical clearance certificates attached as supporting documents. Permission was obtained from District office and health facilities, and the participants signed informed consent before data collection. The participants were not required to put their names in the consent form to maintain anonymity. During recruitment, the participants were given full information about the study including the purpose of the study, and the right to withdraw from participation without any penalty incurred.

## Results and discussion

Two major themes emerged from the observations, namely: facilitators (individual and organisational factors) and inhibitors (information gap, capacity gap, funding gap, policy gap, administrative gap, objective gap, and accountability gap) in guideline implementation.

### Theme 1: Facilitators of enteral nutritional therapy practice guidelines implementation

#### Category 1: Positive attitude and potential conducive practice environment

‘… yeah, we definitely need to have knowledge and skills for that …’ (P1, female, nurse manager in the hospital, 58 years old)

‘Yes, we need to be taught about these things, … and I was excited that we are also feeding patients via a tube at home in South Africa …, it looks interesting …’(P7, female, nurse working in the adult medical/surgical wards, 33 years old)

The observed physical structure of the DH has a potential of providing a comprehensive assessment and rehabilitation of adult patients regarding HEN needs. In other countries, through the development of HEN policies and guidelines, individualised rehabilitation from the time of hospital admission until discharge, is a national quality standard.^8,37,38^

#### Category 2: Good communication culture

There was a pattern in all three institutions of morning gatherings where new documents such as policies and critical clinical cases were discussed. In every department that was observed, the operational managers would be expecting the researcher and would have identified individuals to attend to the information needs.

Across all observed institutions, participants showed positive attitude, amongst themselves, patients and the researcher. They worked in teams, showed etiquette, with effective formal channels of communications:

‘I am surprised to see you today, we were told about you and were expecting you tomorrow, however, I will allocate someone to take you around.’ (P1, female, nurse manager in the hospital, 58 years old)

Communication demonstrated in all these institutions was reported by Ayres and Griffith^39,40^ to be a facilitator for clinical change and guideline adherence. In all three institutions, there was a routine of morning gatherings to share information about patient care and policy.

#### Category 3: Teamwork and collaboration

Again, the hospital was noted to have a complete ideal nutritional therapy team, namely, the dietitian, doctor, pharmacist, professional nurse, speech and language therapist, and other categories included management and support staff. The responses below denote teamwork and collaboration that was observed amongst them. The relationship showed by HCPs had a potential to have a therapeutic effect on HEN patients:^41,42^

‘… talking of which, I think they might have somebody on HEN … we work well together, I can contact them for you or I can give you their number.’ (P2, female, working in nutrition services unit, 32 years old)

‘… I think Miss … will be able to help you she is the speech and language therapist, she is always involved in the nutrition team.’ (P4, female, head of department in rehabilitation unit, 30 years old)

#### Category 4: Management/family support

In all institutions and departments, managers provided support to the researcher by allocating someone relevant to the study to ensure that the researcher got the information she needed. For instance, from the chief executive and Nursing Service Manager’s office, the researcher was referred to the senior doctor, senior pharmacists, area nursing manager, senior dietitian and head of the rehabilitation unit:

‘Our management is very supportive, especially when it comes to issues of growth and development.’ (P6, female, nurse working in the adult medical/surgical wards, 27 years old)

‘My family have been very supportive, you can imagine … I have been feeding like this for more than 10 years now …, the other thing that has kept me going is the support from the hospital X staff. One thing I wish to see happen is getting home visits from the dietitian, instead of me always going there.’ (P12, male, on home enteral nutrition, 19 years old)

#### Category 5: Monitoring culture

During data collection, the researcher personally met three senior members from the health district office on their monitoring rounds in one of the PHC clinics and this is what they had to say:

‘… please remember to give us the results of your study so we can know where we need to improve our service delivery …’ (P10, male, health district office manger, 62 years old)

**FIGURE 3:**
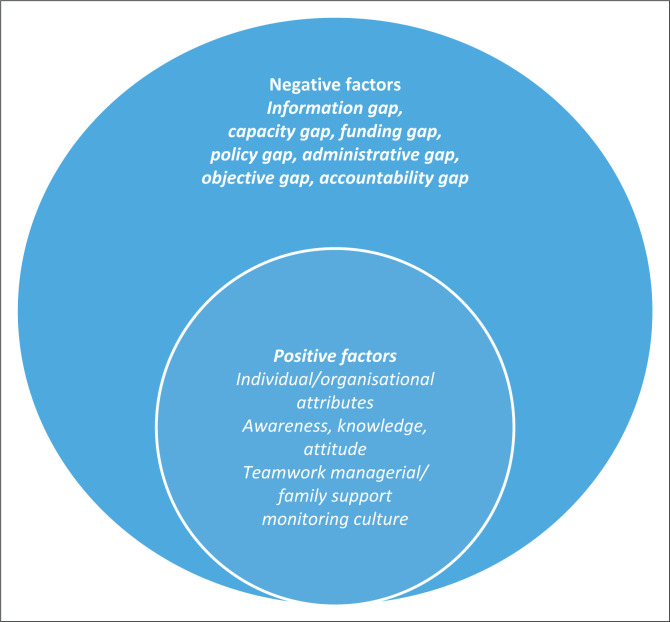
Facilitating and inhibiting factors to enteral nutritional therapy practice guidelines implementation.

It was good to note that monitoring was a norm in the district. Successful monitoring and evaluation system results in improved and relevant policies, a high quality and responsive public service delivery and improved quality of life for all citizens;^43^ hence, the joint effort and input by the District Office Management, Hospital and CHC Management in the district health plan.^27^ Monitoring may help with the identification of residual gaps against the progress made as well as possible interventions to bridge them. For easy reporting, results and themes of the qualitative analysis are presented as Figure 3. Table 1 provides the summary of what was observed.

**TABLE 1:** A summary of what was observed in the physical practice environment.

Setting	ENPGs	Physical environment	Interactions
**A**	Not available	**Physical structure:** Adult wards; male and female surgical and male female medical wards, OPD, dietitians’ office and stoma therapy room attached to OPD, rehabilitation unit next to the medical and surgical wards, gateway clinic in hospital premises in a congested prefabricated building	Etiquette, formal channels of communications, teams routine morning meetings, clinical rounds, case discussions, shared equipment, and rooms which promoted collaboration Culture: Decision-making considered a collaborative process Mandate: Alternative guidelines use mandated, management support from institutional to district level, monthly monitoring visits from district office
		**MDT:** Dietitian, doctor, pharmacist, professional nurse, speech and language therapist	
		**Other:** management and support staff	
		**Equipment and material:** Two feeding pumps in the whole hospital, EN material available	
**B**	Not available	**Physical structure:** Outpatients department with X-ray department and small operating room, adult consulting rooms, treatment room, pharmacy department, nutritionist/dietitian consulting room used for consultation as well as store room	
		**MDT:** Dietitian, doctor, pharmacist, professional nurse	
		**Other:** management and support staff	
		**Equipment and material:** Medication refrigerator, scale to measure weight and height, measuring tape for abdomen, register of patients seen, home visits record book	
**C**	Not available	**Physical structure:** Waiting area, staff office, consulting cubicles, treatment room, kitchen, nutrition/TB/HIV/AIDS counselling cubicle in an outside building, maternity and general sections	
		**MDT:** Professional nurse	
		**Other:** HIV/AIDS and nutrition counsellors	
		**Equipment and material:** Medication refrigerator, scale to measure weight and height, measuring tape for abdomen, register of patients seen, home visits record book	
**D**	Not applicable	**Physical structure:** Three formal rural houses, 1 used as reception room and kitchen, 2 used as bedrooms for a family of 3.	Homely atmosphere, positive attitude, strong family support and acceptance of the condition by family and community
		**Equipment and material:** Old but clean refrigerator to keep food fresh, food blender, a homemade table (working surface to prepare food) and a vegetable stand with vegetables (butternut, potatoes, carrots)	

ENPGs, enteral nutritional therapy practice guidelines; EN, enteral nutritional; OPD, outpatients department; MDT, multidisciplinary team; TB/HIV/AIDS, tuberculosis/human immunodeficiency virus/acquired immunodeficiency syndrome.

### Theme 2: Factors inhibiting guideline implementation

This theme is discussed under seven adopted categories called ‘Mind the gaps’, a diagnosis tool for co-ordination and capacity challenges in the governance of public policies in decentralised contexts as stated by Charbit.^44^

#### Category 2.1: Information gap

According to Charbit,^44^ this type of gap is characterised by information irregularities between levels of government when designing, implementing and delivering public policies. In this study, the participants attested to not having information about the national EN guidelines. Gagliardi, Marshall et al.^45^ speculated that the efforts to publicise guidelines by guideline developers may sometimes be limited by the lack of resources, leading to target users having to take responsibility of implementation:

‘… I don’t know about the new guidelines you are talking about, they must just bring them … yes, we do provide tube feeding … , but we do not have any at the moment … as for PEG [*pe rcutaneous endoscopic gastrostomy*], no we don’t … we get potential candidates but not a single one has ever come back … I don’t know … maybe its transport related …, we sometimes find out that they stay with relatives nearer to referral hospitals and when they come back, they are off it.’ (P2, female, HCP working in nutrition service unit, 32 years old)

Lack of awareness and unavailability of the national ENPGs was observed in all three health institutions, which is consistent with what has been found to be amongst major barriers in scaling up nutritional interventions in South Africa.^46,47,48^ This is a concern and to some extent unacceptable, given the crucial need for actions against illness related malnutrition during recovery and post hospital discharge trajectory.^49^ Vorster, Badham and Venter^47^ emphasised the importance of the provision of evidence-based information in the form of guidelines for the public to understand, relate to and apply.

However, despite the fact that practice guidelines and knowledge are vitally important to evidence-based practice, a gap between the information in the guidelines and the knowledge needed to implement them is a known fact.^50,51^ Additionally, a gap was also noted with regard to down referral system from acute care to community health services. The following excerpts imply poor down referral of patients on HEN from acute health services to DHs and PHC:

‘… I am sure you have seen as you were coming here … our patients travel long distances to come for health services … we lose them along the way.’ (P5, male, nutritionist working in PHC, 29 years old)

‘… I don’t think the guidelines referred to have anything to do with us …, ill patients, when are discharged from hospital don’t come straight to us and when they come they don’t have feeding tubes … even when they do come, who is going to do that here … But how … having them doing at home is worse …, I only read about that … never seen it …’ (P8, female, nurse manager in PHC, 39 years old)

Regarding this, the KZN DoH^27^ attested that transport remains the cost driver in the district in terms of maintenance and repairs resulting from poor landscape, terrains and the distance in-between service points. This may lead to poor patient adherence to nutritional therapy instructions and contribute to poor implementation of ENPGs. However, to mitigate this problem, the KZN report stated that there are Emergency Services and Forensic Services vehicles that are managed through district fleet management. Hopefully, that will also help in HEN services.

#### Category 2.2: Capacity gaps

The capacity gap arises where there is a lack of human resources, knowledge resources or infrastructural resources needed to carry out tasks, regardless of the level of government.^52^ In this study, the participants acknowledged the gaps, but at the same time declared an interest and need for those gaps to be filled.

‘I remember one time I was called to rescue a situation where a patient with a tracheostomy needed suctioning and people were not confident to do it … a family caregiver jumped in saying they were taught how to do it in hospital X …, I had to do a quick in-service training on that … we don’t want the community to lose confidence in us …’ (P1, female, nurse manager in the hospital, 58 years old)

‘… No we don’t do PEGs we just treat and educate patients with colostomies and those with suprapubic catheters …, maybe they are still going to introduce that … even when they do come, who is going to do that here … But how … having them doing at home is worse …, I only read about that … never seen it …’(P9, male, nurse working stoma therapy unit in hospital outpatient’s department, 49 years old)

This following excerpt from participant 12 seems to be confirming what the HCPs were verbalising, which is a concern. Gagliardi, Marshall^45^ viewed organisational capacity as a determinant for the readiness to implement or adopt guidelines:

‘… I do not think that they can help us …, the last time I went there (local clinic) they said they don’t know about PEG [*pe rcutaneous endoscopic gastrostomy*] and they did not have these big syringes … that time I also needed some dressings for this hole around the pipe, that is why I continue to go to hospital X.’ (P12, male, on home enteral nutrition, 19 years old)

#### Category 2.3: Fiscal gap

This is the difference (or gap) between sub-national government revenue and the actual required expenditures for a sub-national government to meet its responsibilities and implement appropriate development strategies.^44,53^ In some dynamic cases, fiscal gaps may also be characterised by the mismatch between budget practices and policy needs. The following extracts are aligned with what Ceronio, Mbhenyane^54^ found in 40 hospitals in Limpopo, South Africa, where, nine, had no employed dietitians:

‘In fact, the last time I checked there were only 30 employed dieticians in the whole country … it is really problem … I don’t know why the government does not employ more because there are hundreds of trained dietitians.’ (P2, female, HCP working in nutrition service unit, 32 years old)

‘… they talk and talk but do not provide us with resources to practice what they say we are doing in paper.’ (P3, male, medical doctor working in hospital medical/surgical wards and rehabilitation unit, 53 years old)

According to the KZN DoH,^27^ in the district there are some of the posts that have been approved in the organogram but are not yet implemented because of the shortage of funds for the filling of the post. The limited working space for dietitians and two feed pumps for the entire institution are an indication of the limited resources, which may contribute to non-compliance to ENPGs. The limited space for dietitians to function effectively could contribute to defaulting of follow-ups and therefore hinder guideline implementation:

‘… This is where the three of us work from …, there is only the three of us for the whole hospital including the gateway clinic, feeder clinics that include the local prison clinic and the government old age home.’ (P2, female, HCP working in nutrition services unit, 32 years old)

‘We do get NG tube fed patients … our challenge is that we have only two feeding pumps for the whole institution [*185 bedded hospital*].’ (P6, female, nurse working in the adult medical/surgical wards, 27 years old)

Despite the available physical structure for HEN in the household, they used a public tap that was located in a central point for the whole neighbourhood, a pit privy and only one room had electricity. There was no one with a permanent job in the family, as such, they had to go bimonthly for follow up care because of financial constraints. The feeding utensils were left uncovered and were not clean. The family care giver stated the following:

‘… It is difficult …, but we are surviving…maybe one day, I will get a permanent job … the challenge is I am not educated …’ (P11, male, family caregiver, 57 years old)

Participant 2 indicated the shortage of human resources, in particular dietitian shortage. This was found consistent with what was reported by Tangvik, Guttormsen et al.^55^ in that they had the lowest number of dietitians in their hospital in all the Western countries. This meant that physicians and nurses had to take over their responsibilities for the patients’ nutritional care. Kraak, Harrigan et al.^56^ recommended that collaboration with private companies and their corporate foundations can help address complex public health nutrition challenges. Another observation made in the selected household of unhygienic practices related to the lack of resources. It could also contribute to complications, readmissions and high care costs to both the state and family.

#### Category 2.4: Policy gap

Government health policy has been implicated as one of the important contextual factors hindering guideline implementation.^57^ This type of gap is said to arise when line departments take a purely vertical approach to local implementation, whilst sub-national governments may be better placed to customise complementariness between policy fields and concretise cross-sectoral approaches.^44^ In order to be cost effective, sub-national governments have to identify relevant paths for local competitiveness and effective service delivery, which largely depends on their institutional background. Limited coordination amongst line ministries may give rise to problems such as administrative overload or differences in timing and agenda in managing correlated actions.^44,58^ McKee et al.^57^ stated that external factors were also implicated and one study^45^ found that the most important barrier to implementation was government health policy. In this study, it was mentioned that they only saw the guidelines on internet:

‘No we do not have guidelines for tube fed patients here and at home … he … eh, maybe they are still going to introduce them … we have an open communication channel with the district office.’ (P8, female, nurse manger in PHC, 39 years old)

#### Category 2.5: Administrative gap

The administrative gap occurs when administration scale for policy making, in terms of spending as well as strategic planning, does not correspond with functionally relevant areas.^44^ This becomes an administrative problem, and leads to disintegration of investment projects at local levels.^59^

#### Category 2.6: Objective gap

The objective gap refers to different rationalities from national and local policymakers leading to resistance in adopting common strategies.^44^ In South Africa, in 2013, stakeholders were seen to have different views on the major causes and the priority of nutritional problems facing the country. The perception was that nutrition action only meant provision of food parcels and food gardens and nothing was mentioned on nutrition as therapy.^31,60^ This study has shown that in 2018, this was still the case. The following statement attests to this fact:

‘you know these politicians are just window dressing, they are mimicking what these successful countries like X are doing but …, but with them it’s just lip service, they do not provide us with resources to practice what they say we are doing in paper.’ (P3, male, medical doctor working in hospital medical/surgical wards and rehabilitation unit, 53 years old)

#### Category 2.7: Accountability gap

This gap is described as a lack of transparency of those liable for disseminating information related to public policy across different constituencies and levels of government.^44^ In South Africa, despite that the guidelines have been developed and put onto the inter/intranet, minimal effort has been made to draw awareness of potential adaptors about their existence. The following quotes are evidence to this:

‘… I have not seen the new guidelines …, they must just introduce them with the necessary resources … we were never even involved in the development of those, how are we going to own them …, we will see.’ (P3, male, medical doctor working in hospital medical/surgical wards and rehabilitation unit, 53 years old)

‘Yes, we need to be taught about these things, I saw the guidelines on the internet and I was excited.’ (P7, female, nurse working in the medical/surgical wards on rotational basis, 33 years old)

A well-functioning accountability infrastructure is recommended to hold stakeholders accountable for the delivery of nutrition services.^61^ The recommendation was based on the observation that accountability is lacking in nutrition in many UN member states.^61^ A summary of this study’s results is presented in Figure 3.

## Methodological strengths and limitations

This study is not without limitations. Direct observation is about reality; it covers events in real time and is contextual as it covers event context. However, its disadvantages include time-consuming and selectivity, which might lead to some facts being missed. Another limitation of this study is that the observer’s presence might have caused change in the observed behaviour. However, to mitigate this, the researcher used ‘hanging out’ to establish rapport with the participants, which resulted in participants becoming familiar with her, acting normally around her and voicing their opinions freely. Furthermore, this type of study approach is always criticised for the lack of objectivity as the researcher serves as a co-constructor of the truth and develops a relationship with the participants, which makes objectivity almost impossible.^23^

## Conclusion and recommendations

According to the researchers’ knowledge, this study is the first to study the implementation of national ENPGs for adults, particularly HEN in the South African context. However, despite the lack of awareness and unavailability of the guidelines and lack of resources, there were positive factors such as institutional or managerial and family support and information sharing culture that were found to have a potential for promoting effective guideline implementation with the provision of implementation resources. In essence, the findings of this study identified that the ENPGs for adults have not been properly or actively disseminated. This study, therefore, may increase the awareness of the guidelines and hopefully, lead to improved implementation thereof. Consequently, the provision of the EN may improve, and complications and readmissions may get reduced, thus saving on health care costs. In addition, the development of a model for the implementation of ENPGs for adults is the ultimate goal. There is a need for more research on this aspect as it is limited in South Africa.

## References

[1] Wischmeyer PE. Tailoring nutrition therapy to illness and recovery. Crit Care. 2017;21(3):316. 10.1186/s13054-017-1906-8 29297385 PMC5751603

[2] Wischmeyer PE. Are we creating survivors or victims in critical care? Delivering targeted nutrition to improve outcomes. Curr Opin Crit Care. 2016;22(4):279–284. 10.1097/MCC.0000000000000332 27327244

[3] Huang J, Yang L, Zhuang Y, Qi H, Chen X, Lv K. Current status and influencing factors of barriers to enteral feeding of critically ill patients: A multicenter study. J Clin Nurs. 2019;28(3–4):677–685. 10.1111/jocn.14667 30182514

[4] Hoffer LJ, Bistrian BR. Nutrition in critical illness: A current conundrum. F1000Res. 2016;5:2531. 10.12688/f1000research.9278.1 27803805 PMC5070594

[5] Heyland DK, Dhaliwal R, Wang M, Day AG. The prevalence of iatrogenic underfeeding in the nutritionally ‘at-risk’ critically ill patient: Results of an international, multicenter, prospective study. Clin Nutr. 2015;34(4):659–666. 10.1016/j.clnu.2014.07.008 25086472

[6] Boullata JI, Carrera AL, Harvey L, et al. ASPEN safe practices for enteral nutrition therapy. JPEN. 2017;41(1):15–103. 10.1177/0148607116673053 27815525

[7] Ojo O. The challenges of home enteral tube feeding: A global perspective. Nutrients. 2015;7(4):2524–2538. 10.3390/nu7042524 25856223 PMC4425159

[8] Gramlich L, Hurt RT, Jin J, Mundi MS. Home enteral nutrition: Towards a standard of care. Nutrients. 2018;10(8):1020. 10.3390/nu10081020 PMC6116140 30081546

[9] Bischoff SC, Austin P, Boeykens K, et al. ESPEN guideline on home enteral nutrition. Clin Nutr. 2020;39(1):5–22. 10.1016/j.clnu.2019.04.022 31255350

[10] Ojo O, Brooke J. Recent advances in enteral nutrition. Nutrients. 2016;8(11):709. 10.3390/nu8110709 PMC5133096 27834792

[11] Holst M, Rasmussen HH. Nutrition therapy in the transition between hospital and home: An investigation of barriers. J Nutr Metabol. 2013;2013:463751. 10.1155/2013/463751 PMC3893861 24490060

[12] Faruquie SS, Parker EK, Talbot P. An evaluation of current home enteral nutrition services at principal referral hospitals in New South Wales, Australia. Aust Health Rev. 2016;40(1):106–113. 10.1071/AH15029 26235412

[13] McCall ME, Adamo A, Latko K, Rieder AK, Durand N, Nathanson T. Maximizing nutrition support practice and measuring adherence to nutrition support guidelines in a Canadian Tertiary Care ICU. J Intensive Care Med. 2017;33(3):209–217. 10.1177/0885066617749175 29284322

[14] Tapp H, White L, Steuerwald M, Dulin M. Use of community-based participatory research in primary care to improve health care outcomes and disparities in care. J Comp Eff Res. 2013;2(4):405–419. 10.2217/cer.13.45 PMC4042315 24236682

[15] Department of Health. A long and healthy life for All South Africans: National enteral nutrition practice guidelines for adults. In: Health Do, editor. Pretoria: Department of Health; 2016.

[16] Blaauw R. Diagnosis of hospital malnutrition in the adult population. S Afr J Clin Nutr. 2019;32(1):8–10.

[17] World Health Organization. Meeting report: WHO technical consultation: Nutrition-related health products and the World Health Organization model list of essential medicines–practical considerations and feasibility. Geneva, Switzerland: WHO. 20–21 September 2018. World Health Organization; 2019.

[18] WHO. Regional strategy on nutrition 2010–2019 and plan of action. Egypt: WHO; 2011.

[19] WHO. The double burden of malnutrition. Policy brief. Geneva: World Health Organization; 2017.

[20] WHO. Global database on the Implementation of Nutrition Action (GINA). World Geneva: Health Organization; 2012.

[21] Van Tonder E, Gardner L, Cressey S, Tydeman-Edwards R, Gerber K. Adult malnutrition: Prevalence and use of nutrition-related quality indicators in South African public-sector hospitals. S Afr J Clin Nutr. 2019;32(1):1–7. 10.1080/16070658.2017.1410003

[22] Blomberg J, Giacomi J, Mosher A, Swenton-Wall P. Ethnographic field methods and their relation to design. Participatory design. Florida: CRC Press, 2017; p. 123–155.

[23] Kawulich BB, editor. Participant observation as a data collection method. Forum Qualitative Sozialforschung/Forum: Qualitative Social Research. Georgia: Scientific Research; 2005.

[24] Ritchie J, Lewis J, Nicholls CM, Ormston R. Qualitative research practice: A guide for social science students and researchers. New Delhi: Sage; 2013.

[25] Pandey J. Deductive approach to content analysis. Qualitative techniques for workplace data analysis. Hyderabad, Telangana: IGI Global, 2019; p. 145–169.

[26] Silverman D. Qualitative research. Chennai: Sage; 2016.

[27] KZN DoH. Department of Health KwaZulu-Natal province (2019). Harry Gwala district health plan 2018/19–2020/21 (Kwazulu-Natal). In: Health Do, editor. Pretoria: Government Printers; 2019.

[28] Lincoln YS. Naturalistic inquiry. The Blackwell encyclopedia of sociology. Hoboken, NJ: Wiley Online Library; 1985.

[29] Morse JM. Critical analysis of strategies for determining rigor in qualitative inquiry. Qual Health Res. 2015;25(9):1212–1222. 10.1177/1049732315588501 26184336

[30] Merriam SB, Tisdell EJ. Qualitative research: A guide to design and implementation. Chennai: John Wiley & Sons; 2015.

[31] Department of Health. Roadmap for Nutrition in South Africa 2013–2017. In: Health Do, editor. ZAF 2013 Roadmap for Nutrition in South Africa 2013-2017-1-50. Pretoria: Department of Health; 2013.

[32] Elo S, Kyngäs H. The qualitative content analysis process. J Adv Nurs. 2008;62(1):107–115. 10.1111/j.1365-2648.2007.04569.x 18352969

[33] Graneheim UH, Lundman B. Qualitative content analysis in nursing research: Concepts, procedures and measures to achieve trustworthiness. Nurse Educ Today. 2004;24(2):105–112. 10.1016/j.nedt.2003.10.001 14769454

[34] Erlingsson C, Brysiewicz P. A hands-on guide to doing content analysis. Afr J Emerg Med. 2017;7(3):93–99. 10.1016/j.afjem.2017.08.001 30456117 PMC6234169

[35] Guba EG, Lincoln YS. Fourth generation evaluation. California: Sage; 1989.

[36] Penner JL, McClement S, Lobchuk M, Daeninck P. Family members’ experiences caring for patients with advanced head and neck cancer receiving tube feeding: A descriptive phenomenological study. J Pain Symptom Manag. 2012;44(4):563–571. 10.1016/j.jpainsymman.2011.10.016 22699088

[37] Walsh EG, Wiener JM, Haber S, Bragg A, Freiman M, Ouslander JG. Potentially avoidable hospitalizations of dually eligible medicare and medicaid beneficiaries from nursing facility and home-and community-based services waiver programs. J Am Geriatr Soc. 2012;60(5):821–829. 10.1111/j.1532-5415.2012.03920.x 22458363

[38] Bischoff SC, Austin P, Boeykens K, et al. ESPEN guideline on home enteral nutrition. Clin Nutr. 2019;39(1):5–22. 10.1016/j.clnu.2019.04.022 31255350

[39] Ayres CG, Griffith HM. Perceived barriers to and facilitators of the implementation of priority clinical preventive services guidelines. Am J Manag Care. 2007;13(3):150–156. 17335358

[40] Mendoza Lemire A, Miles A, McCann CM. Changing clinical practice: Facilitators and barriers to the implementation of a nationwide videofluoroscopy evidence-based guideline. Speech Lang Hear. 2016;19(2):69–78. 10.1080/2050571X.2015.1101893

[41] Herridge MS, Tansey CM, Matté A, et al. Functional disability 5 years after acute respiratory distress syndrome. New Engl J Med. 2011;364(14):1293–1304. 10.1056/NEJMoa1011802 21470008

[42] Dowdy DW, Eid MP, Sedrakyan A, et al. Quality of life in adult survivors of critical illness: A systematic review of the literature. Intensive Care Med. 2005;31(5):611–620. 10.1007/s00134-005-2592-6 15803303

[43] Abrahams MA. A review of the growth of monitoring and evaluation in South Africa: Monitoring and evaluation as a profession, an industry and a governance tool. Afr Eval J. 2015;3(1):8. 10.4102/aej.v3i1.142

[44] Charbit C. Governance of public policies in decentralised contexts. OECD Regional Development Working Papers, 2011/04. Paris: OECD Publishing; 2011. 10.1787/5kg883pkxkhc-en

[45] Gagliardi AR, Marshall C, Huckson S, James R, Moore V. Developing a checklist for guideline implementation planning: Review and synthesis of guideline development and implementation advice. Implement Sci. 2015;10:19. 10.1186/s13012-015-0205-5 25884601 PMC4329197

[46] Mooi NM, Ncama BP. Perceived potential barriers to implementation of nutritional therapy practice guidelines in critically ill adults in a district of KwaZulu-Natal, South Africa. Global J Health Sci. 2019;11(11):1–42. 10.5539/gjhs.v11n11p42

[47] Vorster HH, Badham J, Venter C. An introduction to the revised food-based dietary guidelines for South Africa. S Afr J Clin Nutr. 2013;26(3):S5–S12.

[48] Tickell KD, Mangale DI, Tornberg-Belanger SN, et al. A mixed method multi-country assessment of barriers to implementing pediatric inpatient care guidelines. PLoS One. 2019;14(3):e0212395. 10.1371/journal.pone.0212395 30908499 PMC6433255

[49] Kotlowitz JR. Assessment of the implementation of the peri-operative nutrition ERAS guidelines in elective colorectal surgery patients in a tertiary hospital in South Africa. Stellenbosch: Stellenbosch University; 2017.

[50] Baradaran-Seyed Z, Nedjat S, Yazdizadeh B, Nedjat S, Majdzadeh R. Barriers of clinical practice guidelines development and implementation in developing countries: A case study in Iran. Int J Prev Med. 2013;4(3):340. 23626892 PMC3634174

[51] Shiffman RN, Michel G, Essaihi A, Thornquist E. Bridging the guideline implementation gap: A systematic, document-centered approach to guideline implementation. J Am Med Informat Assoc. 2004;11(5):418–426. 10.1197/jamia.M1444 PMC516249 15187061

[52] Charbit C, Michalun MV. Mind the gaps: Managing mutual dependence in relations amongst levels of government. OECD Working Papers on Public Governance, No. 14. Paris: OECD Publishing; 2009. 10.1787/221253707200

[53] Ackah I, Osei E, Tuokuu FXD, Bobio C. Oiling the wheels of sub-national development: An overview of development plan implementation in the Western region of Ghana. Extractive Indust Soc. 2019;6(2):343–357. 10.1016/j.exis.2018.12.002

[54] Ceronio V, Mbhenyane XJJoN, Health. Dietary management practices for diabetes by dietitians in public hospitals in Limpopo Province, South Africa. J Nutr Health. 2017;3(1):1–6.

[55] Tangvik RJ, Guttormsen AB, Tell GS, Ranhoff AH. Implementation of nutritional guidelines in a university hospital monitored by repeated point prevalence surveys. Eur J Clin Nutr. 2012;66(3):388–393. 10.1038/ejcn.2011.149 21863042 PMC3303136

[56] Kraak VI, Harrigan PB, Lawrence M, Harrison PJ, Jackson MA, Swinburn B. Balancing the benefits and risks of public–private partnerships to address the global double burden of malnutrition. Publ Health Nutr. 2012;15(3):503–517. 10.1017/S1368980011002060 22014282

[57] McKee G, Kerins M, Hamilton G, et al. Barriers to ESC guideline implementation: Results of a survey from the European Council on Cardiovascular Nursing and Allied Professions (CCNAP). Eur J Cardiovasc Nurs. 2017;16(8):678–686. 10.1177/1474515117710097 28498092

[58] Wu X, Ramesh M, Howlett M, Fritzen SA. The public policy primer: Managing the policy process. Oxfordshire: Routledge; 2017.

[59] Cirolia LR. Fractured fiscal authority and fragmented infrastructures: Financing sustainable urban development in sub-Saharan Africa. Habitat Int. 2020;104:102233. 10.1016/j.habitatint.2020.102233

[60] Du Plessis LMJSAJoCN. Commitment and capacity for the support of breastfeeding in South Africa: A paediatric food-based dietary guideline. S Afr J Clin Nutr. 2013;26:S120–S128.

[61] Haddad L, Achadi E, Bendech MA, et al. The Global Nutrition Report 2014: Actions and accountability to accelerate the world’s progress on nutrition. J Nutr. 2015;145(4):663–671. 10.3945/jn.114.206078 25740908 PMC5129664

